# Quantifying rooting at depth in a wheat doubled haploid population with introgression from wild emmer

**DOI:** 10.1093/aob/mcx068

**Published:** 2017-06-26

**Authors:** Christina K Clarke, Peter J Gregory, Martin Lukac, Amanda J Burridge, Alexandra M Allen, Keith J Edwards, Mike J Gooding

**Affiliations:** 1 School of Agriculture, Policy and Development, University of Reading, Earley Gate, PO Box 237, Reading RG6 6AR, UK,; 2 Czech University of Life Sciences, 16521 Prague, Czech Republic,; 3 Life Sciences, University of Bristol, Bristol, Avon BS8 1TQ, UK and; 4 Institute of Biological, Environmental and Rural Sciences, University of Aberystwyth, Gogerddan, Aberystwyth, Ceredigion SY23 3EE, UK

**Keywords:** Deep rooting, drought, phenotyping, root length density, doubled haploid, seedling screen, rhizotron, Triticum aestivum (wheat), Triticum dicoccoides (wild emmer)

## Abstract

**Background and Aims:**

The genetic basis of increased rooting below the plough layer, post-anthesis in the field, of an elite wheat line (*Triticum aestivum* ‘Shamrock’) with recent introgression from wild emmer (*T. dicoccoides*), is investigated. Shamrock has a non-glaucous canopy phenotype mapped to the short arm of chromosome 2B (2BS), derived from the wild emmer. A secondary aim was to determine whether genetic effects found in the field could have been predicted by other assessment methods.

**Methods:**

Roots of doubled haploid (DH) lines from a winter wheat (‘Shamrock’ × ‘Shango’) population were assessed using a seedling screen in moist paper rolls, in rhizotrons to the end of tillering, and in the field post-anthesis. A linkage map was produced using single nucleotide polymorphism markers to identify quantitative trait loci (QTLs) for rooting traits.

**Key Results:**

Shamrock had greater root length density (RLD) at depth than Shango, in the field and within the rhizotrons. The DH population exhibited diversity for rooting traits within the three environments studied. QTLs were identified on chromosomes 5D, 6B and 7B, explaining variation in RLD post-anthesis in the field. Effects associated with the non-glaucous trait on RLD interacted significantly with depth in the field, and some of this interaction mapped to 2BS. The effect of genotype was strongly influenced by the method of root assessment, e.g. glaucousness expressed in the field was negatively associated with root length in the rhizotrons, but positively associated with length in the seedling screen.

**Conclusions:**

To our knowledge, this is the first study to identify QTLs for rooting at depth in field-grown wheat at mature growth stages. Within the population studied here, our results are consistent with the hypothesis that some of the variation in rooting is associated with recent introgression from wild emmer. The expression of genetic effects differed between the methods of root assessment.

## INTRODUCTION

There is a challenge to improve wheat yield and yield stability while the amplitude and frequency of weather fluctuations increase (Hansen *et al.*, 2012; Ray *et al.*, 2015). Climate projections predict rising temperatures and reduced summer rainfall in major wheat-growing areas, in the UK and globally (Jenkins *et al.*, 2010). A large proportion of the world’s wheat crop is rainfed, and water deficits from meiosis onwards reduce wheat productivity, as both grain set and grain filling are compromised (Gooding *et al.*, 2003; Barber *et al.*, 2015). Crop and field management influences the availability of soil water and soluble nutrients (Thorup-Kristensen and Kirkegaard, 2016), but deep rooting can also improve access to sub-soil water (Wasson *et al.*, 2012; Lilley and Kirkegaard, 2016). Wheat roots can potentially extend to 2 m below the soil surface (Thorup-Kristensen *et al.*, 2009). In one experiment, the 3 % of the total root system found below 1 m supplied nearly 20 % of the water evaporated during grain filling (Gregory *et al.*, 1978b).

Wheat improvement in breeding programmes of modern cultivars has focused on above-ground biomass accumulation and partitioning. In contrast, potential yield improvements through more efficient biomass allocation in root systems have not been explored in detail (Smith and De Smet, 2012). Breeding of varieties released since the 1960s, as part of the so-called green revolution, focused on above-ground traits and increasing the harvest index. This may have reduced root biomass at maturity by>50 % compared with landrace wheats (Waines and Ehdaie, 2007). A reduction in root biomass may arise through an unconscious selection of root systems sufficient for good or near optimal growing systems but which may increase the vulnerability of the crop to heat or drought stress. Root length density (RLD) of modern UK wheats is considered inadequate for water uptake in the sub-soil because RLDs of<1cm cm^−3^ are frequently recorded below 40cm depth (Hoad *et al.*, 2004). A threshold of 1cm cm^−3^ has been determined as the critical RLD sufficient to acquire water and nitrogen resources (van Noordwijk, 1983; Barraclough *et al.*, 1989; White *et al.*, 2015). Greater investment in fine roots at depth, at the expense of roots close to the surface, would be more efficient for acquiring water and solutes such as nitrate (King *et al.*, 2003). Uptake of stored sub-soil water can be particularly effective at stabilizing yields if the duration of grain growth and retranslocation processes can be maintained while a terminal drought develops after meiosis (Kirkegaard *et al.*, 2007; Wasson *et al.*, 2012).

The high plasticity and low heritability of root system traits makes them difficult to study and therefore select for in breeding systems (de Dorlodot *et al.*, 2007). Dry soil, coupled with the large size of the root system compared with early growth stages, means that the quantification of RLD at depth is particularly challenging during the later growth stages when terminal droughts are likely to develop. Nonetheless, an elite line of winter wheat, *Triticum aestivum* ‘Shamrock’, was found repeatedly to exhibit greater root length below a 30cm deep ploughed layer in UK field plots at and after anthesis (Ford *et al.*, 2006). Shamrock has a notable pedigree: the cultivar was first recommended in the late 1990s and contains material recently introgressed from wild emmer (*Triticum dicoccoides*); derived from a cross between NW Europe germplasm (CWW 4899/25 – Moulin × Monopol) and a *T. dicoccoides* derivative (Comp Tig 323-1-3 M) (Simmonds *et al.*, 2008). Wild relatives of wheat can provide a valuable germplasm resource for improving yield performance under abiotic stress, due to their origin and diversification in dry environments such as the Fertile Crescent (Xie and Nevo, 2008; Nevo and Chen, 2010). Shamrock inherited a non-glaucous trait, the reduction of epicuticular wax, through the wild emmer introgression, which is apparent at flag leaf emergence. The trait is associated with delayed senescence and causes a striking green colour of the canopy. The non-glaucous gene has been mapped to the short arm of chromosome 2B (Simmonds *et al.*, 2008), associated with the wax inhibitor gene *Iw1* which reduces the β-diketone component of the plant wax (Adamski *et al.*, 2013).

We are unaware of previous studies that have succeeded in identifying quantitative trait loci (QTLs) among well-adapted elite germplasm that associate with differences in root traits in the field at depth, late in the growing season. The primary aim of this study was to use a doubled haploid (DH) mapping population to investigate the genetic basis for improved rooting at depth of ‘Shamrock’. Of particular interest were potential associations of single nucleotide polymorphism (SNP) markers on 2BS and the non-glaucous phenotype from wild emmer, with RLD. Further, due to the challenges of washing and assessing roots from soil cores taken from the field at anthesis, we investigated the potential utility of assessing early root system growth in a seedling screen (Bai *et al.*, 2013; Watt *et al.*, 2013) and within 1 m tall rhizotrons (Liao *et al.*, 2006) to predict deep rooting in the field.

## MATERIALS AND METHODS

### Plant material and genetic mapping

Eighty-seven lines of the Shamrock × Shango DH population, and the two parents, (Simmonds *et al.*, 2008) were genotyped using the Axiom^®^ Wheat Breeder’s 35k Genotyping Array (Affymetrix Inc., Santa Clara, CA, USA; Allen *et al.*, 2017), and Kompetitive Allele Specific PCR (KASP™) genotyping chemistry (LGC Ltd, Teddington, UK; Allen *et al.*, 2011). A linkage map containing 21 groups was produced using 3785 SNP markers and MapDisto 2.0 software (Lorieux, 2012). Total map length is 3126cM, with an average linkage group length of 148cM and a median distance between markers of 2·3cM. Linkage groups were determined using a logarithm of odds (LOD) threshold of 3.0 and a recombination fraction of 0·3. Genetic distances were computed using the Kosambi (1943) mapping function, and markers with significant segregation distortion values were removed from the map, assessed using the χ^2^ test. SNPs were anchored to chromosomes using the consensus genetic map produced by merging five genetic maps from mapping populations genotyped using the Wheat Breeder’s 35k Array (Allen *et al.*, 2017). Duplicated markers were removed from the map based on agreement with the consensus map and previously genetically mapped SNPs from CerealsDB (Wilkinson *et al.*, 2012). If a marker matched the position in the survey sequence it was retained, but if no previous position was defined it was removed. Duplicates were removed at random if both agreed with the survey sequence position.

### Field experiment

Two randomized blocks containing each DH line were sown at Reading University Crops Research Unit, Sonning, UK (0°54′W, 51°29′N) in each of the 2013/2014 and 2014/2015 growing seasons (15 October 2013 and 25 September 2014); the parents were sown twice in each block. The field was power harrowed after ploughing to a nominal depth of 30cm. Seeds were drilled in 2 × 5 m^2^ plots at 300 seeds m^−2^ in 120mm rows on a free draining sandy loam overlying coarse red-brown sand (Sonning series; Jarvis, 1968). In the first year, the wheat was the third cereal following a grass ley; in the second year the wheat was the first cereal after a 3year grass plus clover ley. All plots received 16kg S ha^−1^ in both years, and 200kg N ha^−1^ in 2013/14 and 235kg N ha^−1^ in 2014/15 as granular fertilizer during stem extension. Weeds and foliar pathogens were adequately controlled with standard herbicides and fungicides. Weather was recorded in both seasons using an on-site weather station. Average temperatures for winter (October–February) were 7·5°C for 2013/2014 and 6·8°C for 2014/2015, and for stem extension (March–May) were 10·5°C for 2014 and 9·4°C for 2015. Total rainfall for winter was 494mm (2013/2014) and 328mm (2014/2015), and for stem extension was 163mm (2014) and 80mm (2015). The plots were harvested using a combine harvester, and grain yield was determined.

Photosynthetic active radiation (PAR) interception was measured throughout the growing season. A ceptometer (AccuPAR LP-80; Decagon Devices Inc., Pullman WA, USA) was used to measure PAR above and below the canopy. Interception of PAR during the crop growth cycle was calculated as in Gooding *et al.* (2002). The end of canopy photosynthetic function was determined as the onset of rapid senescence which coincides with the point of 80 % maximum green cover, calculated as in Addisu *et al.* (2010). Green cover was assessed by measuring the far red (730nm) and red (660nm) reflectance wavelengths with sensors (SKR 1800, Skye Instruments Ltd, Llandrindod Wells, UK), also throughout crop growth. The mean of three readings per plot on each assessment date were used for these canopy assessments.

Roots were sampled during the first 3weeks after anthesis (growth stage (GS) 63: 2 June 2014 and 15 June 2015). In 2014, only the Shamrock and Shango parents were sampled to a depth of 70cm using a steel hand corer of 80mm diameter. Cores were split into 15cm sections in the top 30cm (the plough layer) and 10cm thereafter (below the plough layer). Each core section was placed in a sealed bag and stored in a cold room (2–4°C) prior to washing. Five cores were taken between the rows and three cores within the row due to multiple studies stating that cores solely taken within the row, in addition to small auger sizes, can overestimate real RLDs (van Noordwijk *et al.*, 1985; Kumar *et al.*, 1993; Buczko *et al.*, 2009). A pumped root washing system was used to separate roots from the soil over a 550μm mesh collection filter (Root Washer, Delta T, Cambridge, UK). Roots were hand separated from organic debris: washed samples from the top 30cm were sub-sampled due to large amounts of debris. Roots were then scanned using a flatbed scanner (Expression 1600 XL-PRO, Epsom UK Ltd) at 300dpi resolution and assessed using ‘WinRhizo’ (Regents Instruments Inc., QC, Canada). Samples were dried at 80°C for 48h and weighed. In 2015, roots from all lines were measured in soil collected between 50 and 80cm depths. These samples were collected with a 73mm diameter window sampler driven into the ground using a tractor-mounted hydraulic static pile driver (model number MCL2, Norsk Hydro, Geonor, Norway). Three samples were taken per plot: two between the row and one within the row. Samples were analysed as in 2014 after splitting cores into 10cm sections (50–60, 60–70 and 70–80cm).

### Seedlings

A paper roll system was used to grow the DH population to seedling stage, as described by Bai *et al.* (2013). Seeds of uniform mass (0·05 ± 0·005g) were surface-sterilized in 0·5 % calcium hypochlorite [Ca(ClO)_2_] solution for 30min before being rinsed with sterilized water and then placed in a cold room at 4°C overnight. Seeds were then pre-germinated at 10°C on paper wetted with sterilized water for 72h. Three pre-germinated seeds from each line were placed in a roll of germination paper (Anchor Paper Company, Saint Paul, MN, USA) 2cm wide and 38cm tall. Germination paper rolls were supported within a wire lattice on a tray of nutrient solution in four randomized blocks, giving 12 replicates of each DH line (Bai *et al.*, 2013). Half-strength solution was used for the first 3 d and thereafter replaced with full-strength solution, which was changed every day, for the remainder of the experiment. The trays were placed in a controlled-environment cabinet [12h day, light intensity 500μmol m^−2^ s^−1^, 70 %/80 % day/night relative humidity 20°C/16°C day/night temperatures (Bai *et al.*, 2013)]. After 11 d, the paper rolls were placed in sealed plastic bags and stored at 3°C until root analysis. The whole experiment was repeated to achieve 24 replications per line.

Seedlings were removed from the roll and separated into root and shoot. Intact root systems were scanned (as above) and assessed with ‘WinRhizo’ to obtain: total root length, root surface area, root volume, average diameter and percentage of root length in diameter classes 0–0·5mm and 0·5–1mm. Additionally, the number of seminal axes was counted. Roots and shoots were dried at 80°C for 48h and then weighed so that the root:shoot ratio could be calculated.

### Rhizotrons

Nineteen lines were selected based on total root length, average diameter and root dry weight measured in the seedling experiment, to capture observed root trait variation in glaucous and non-glaucous lines, including parents Shamrock and Shango. A wild emmer accession, obtained from the John Innes Centre Germplasm Resource Unit, was also included to give a comparison of rooting traits in a wild relative.

The lines were grown in 1 m tall × 0·3 m wide × 0·05 m deep root observation chambers (rhizotrons) constructed from PVC sheets with a clear acrylic sheet bolted onto the front of the box (adapted from Liao *et al.*, 2006). Four 8mm diameter holes were drilled into the base and a thin layer of 10mm gravel was placed at the bottom of each rhizotron to aid drainage. A loamy sand (Sporting Surface Supplies Ltd, Smallfield, UK) of composition 40 % sand, 40 % silt and 20 % clay was sieved to 6mm and packed to a bulk density of 1·2g cm^−3^; the soil had 20 % moisture when packed. Rhizotrons were individually wrapped in thermawrap silver foil to insulate them and to ensure the soil was not exposed to light. Seeds of uniform mass (0·05 ± 0·005g) were pre-germinated as for the seedling experiment. Four germinated seeds were sown in a row close to the clear acrylic front of each rhizotron and thinned to two plants per rhizotron after 8 d. The equivalent of 50kg N ha^−1^ as urea and 8kg P ha^−1^ as superphosphate was applied on the soil surface in a solid form. The rhizotrons were placed at a 30 ° angle from vertical against a steel frame, to allow the roots to grow against the clear acrylic front, and spaced 0·10 m apart. Rhizotrons of each genotype were replicated in three randomized blocks in a naturally lit glasshouse in the spring of 2015 at the University of Reading UK. Wheat plants were grown to GS 29 (Zadoks *et al.*, 1974) when roots had reached the 1 m deep base in 50 % of rhizotrons, 45 days after sowing (DAS). Average daily temperatures during the growth period ranged from a minimum of 11°C to a maximum of 25°C, with a natural photoperiod of about 12h. Adequate moisture was provided by watering every 3 d.

From 10 DAS, roots were manually traced twice a week on to acetate sheets taped to the front of each rhizotron. All data originating from the acetate sheets are reported in cm cm^−2^ and denote unit root length per unit surface area of the rhizotron. On 45 DAS (GS 29), the shoots were removed from the crown and dried at 80°C for 48h. The soil and roots in each rhizotron were collected in 0·2 m depth intervals and stored at 2–5°C until the roots were sieved from the soil using a 4mm mesh sieve. Sieved root samples and acetate sheets were scanned as described above with ‘WinRhizo’. Finally, root samples were weighed after drying at 80°C for 48h.

### Statistical analysis

For the field experiment, an attempt was made to control error variation within the blocks by including row and column of the plot positions as incomplete blocks within an analysis of residual maximum likelihood [REML; Genstat v15 (VSN International)], i.e. the random model was Block/(Row + Column) for canopy measurements and roots collected in 2014. Block/(Row + Column)/Plot/Core/Depth was the random model for roots collected in 2015. The fixed model was Line, and adjusted ‘means’ were calculated (best linear unbiased predictors). The interaction of RLD with depth was assessed using Depth × Line as the fixed model. For the seedling experiment, Line means and errors were calculated within an analysis of variance (ANOVA) combining both replicate experiments (block structure: Replicate experiment/Block). The correlation matrix of the seedling variate × Line means was used in a principal component analysis (PCA). For the rhizotron experiment, two ANOVAs were conducted; the first with a treatment structure of Line and block structure of Block and the second ANOVA with glaucousness as a fixed effect within a nested treatment structure of Species/Glaucousness, where Species is either wild emmer or bread wheat.

The line means from seedling and field experiments were used in a QTL analysis with composite interval mapping (CIM) in Windows QTL Cartographer version 2.5 (Wang *et al.*, 2010). Standard model 6 with the forward and backward regression method was used. Ten control markers were automatically selected; window size was set at 10cM and a walk speed setting of 1cM was used for the analysis. LOD threshold values (*P*<0·05) were set by running 1000 permutations to identify significant QTLs. Due to the glaucous trait being binary, Multiple Interval Mapping (MIM) was used to identify QTLs for this trait with the same thresholds (Li *et al.*, 2006).

## RESULTS

### Genetic diversity in the doubled haploid population for rooting at depth in the field

**Fig. 1.**
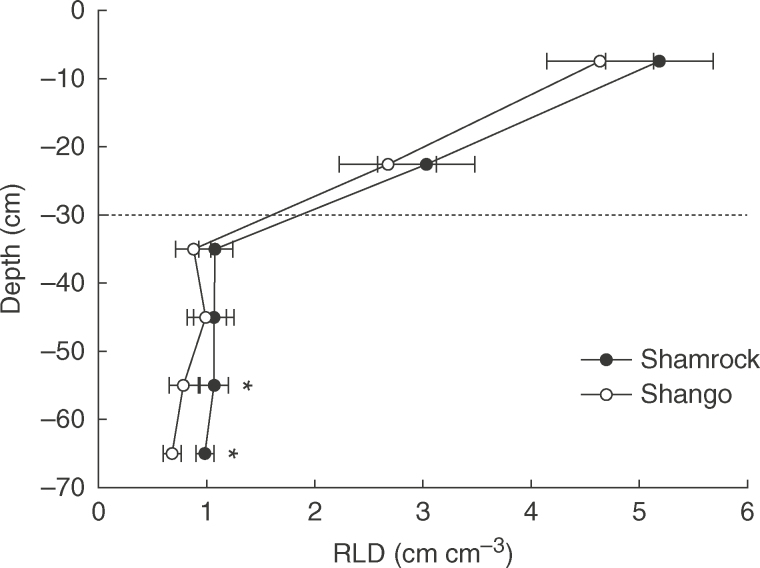
Root length density (RLD) of field-grown Shamrock and Shango winter wheat at anthesis in 2014. Average of 32 cores per genotype. Error bars are ± SED, *P<0·05. The dotted line indicates the plough layer.

**Fig. 2.**
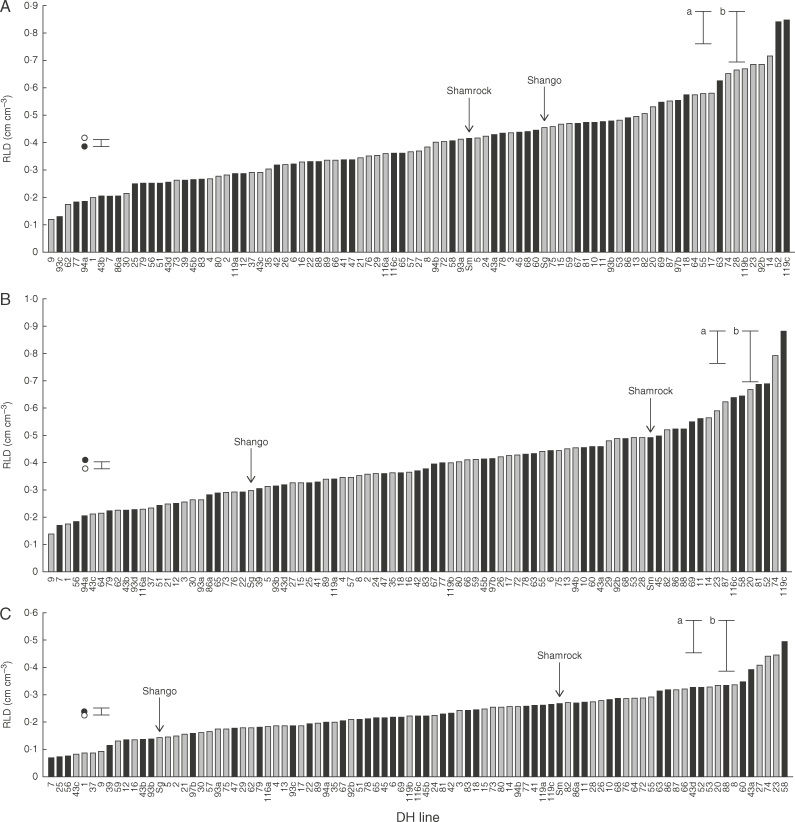
Root length densities (RLDs) of a Shamrock × Shango doubled haploid (DH) population in field-grown plots at anthesis. (A) 50–60cm depth; (B) 60–70cm depth; (C) 70–80cm depth. Black bars denote non-glaucous lines and grey bars glaucous lines. Shamrock and Shango means are labelled. Circles represent means of glaucous (open circles) and non-glaucous (filled circles) lines. The error bar is 1 SED for line means and glaucous and non-glaucous average; (a) SED for Shamrock × Shango, *n*=12; and (b) SED for DH line, *n*=6.

The decline in RLD with depth observed in 2014 (Fig. 1) is typical for the site (Ford *et al.*, 2006), with the plough depth presenting a notable demarcation. Shamrock had consistently higher RLD than Shango below the plough layer; significantly so (*P*<0·05) in the 50–60 and 60–70cm layers. Shamrock also had significantly greater root dry weight (RDW) in the 60–70cm layer [Shamrock=0·0063mg cm^−3^, Shango=0·0044mg cm^−3^, standard error of the difference (SED) 0·00051]. In 2015, significant differences (*P*<0·001) were found between DH lines for RLD and RDW in the 50–80cm layer at anthesis (split into 10cm sections of 50–60, 60–70 and 70–80cm; Fig. 2). The RLD values averaged for the 50–80cm layer ranged from 0·116 to 0·660cm cm^−3^, with no line exceeding 1cm cm^−3^ (SED 0·1401). Maximum rooting depth was not measured in this study, but rooting has previously been reported below 1 m at this site (Gregory *et al.*, 2005). However, gravel below 0·8 m severely restricted both sampling and rooting depths in the present study. The DH lines which consistently performed well within the 10cm soil layers for RLD between 50 and 80cm were 23, 52, 58, 119c, 74, 14 and 20. Consistently low performing lines were 9, 1, 7, 56, 43b, 62 and 94a. The RLD and RDW were positively correlated (*r*=0·82, *P*<0·001). RDW values ranged from 0·004 to 0·045mg cm^−3^ (SED 0·0099). Lines differed significantly (*P*<0·02) for mean root diameter in the 50–80cm layer, ranging from 0·202 to 0·252mm (SED 0·0141). Shamrock had significantly (*P*<0·02) finer roots than Shango (0·216 and 0·238mm, respectively; SED 0·0099). Diameter correlated negatively with RLD (*r*=–0·33, *P*<0·01).

The RLD and RDW of DH lines also differed significantly with depth in the 50–80cm soil core (*P*<0·001; RLD shown in Fig. 2). The location of the non-glaucous gene was confirmed on the short arm of 2B (Table 1; Simmonds *et al.*, 2008). Non-glaucous lines had delayed senescence (*P*<0·001, non-glaucous=2389 and glaucous=2363°Cd; SED 5·46) and greater light interception (*P*<0·001, non-glaucous=631·2 and glaucous=610·9MJ m^−2^; SED 5·18) compared with glaucous DH lines over the growing season, averaged over the two field trials. The DH lines flowered within 2–3 d of each other; timing of anthesis, therefore, is not expected to have affected time to senescence observations. QTLs were identified for these canopy characteristics on 2BS (Table 1), co-locating with glaucousness. Glaucousness did not show an association with average RLD or RDW in the field over the whole 50–80cm core in the DH population. However, the interaction of RLD with soil depth and glaucousness was significant (*P*=0·01), because the mean ‘effect’ of glaucousness at 50–60cm depth contrasted with that at 60–70cm depth (Fig. 2). Consequently, a QTL was identified on 2BS, in close proximity to the glaucous QTL, explaining variation in RLD within the 50–60cm soil layer, with Shango contributing the high value allele (Table 1). Non-glaucousness associated with higher grain yields (*P*=0·02, effect=+0·43 t ha^−1^; SED 0·186), when averaged over the two field seasons.

**Table 1.** Quantitative trait loci (QTLs) from a Shamrock × Shango doubled haploid population for glaucousness, thermal time to senescence (ttSen, °Cd), photosynthetic light absorbed over the field season (PAR, MJ m^−2^), root length density (RLD, cm cm^−3^), root dry weight (RDW, mg cm^−3^) and root diameter (Diam, mm)

Trait	QTL	Chromosome	Position (cM)	Confidence interval (cM)	LOD	Peak marker	Additive effect	Variation explained (%)
Glaucousness	qGlcsns.f-2B	2B	1·0	0–2·1	58·5	BS00084668	0·489	95·6
PAR	qPAR.f-2B	2B	4·7	2·1–6·9	6·5	AX-94939920	10·31	19·6
ttSen	qttSen.f-2B	2B	3·5	0·0–6·2	8·4	AX-94777767	12·60	19·4
RLD	qRLD.f-5D	5D	31·3	18·5–39·5	3·1	BS00158384	0·0355	8·4
RLD	qRLD.f-6B	6B	73·1	66·4–81·9	4·3	AX-94475756	0·0431	13·9
RLD	qRLD.f-7B	7B	12·8	11·1–22·8	3·4	AX-94826552	–0·0342	8·8
RDW	qRDW.f-2A	2A	41·5	38·5–45·1	3·3	AX-94604266	–0·0026	8·7
RDW	qRDW.f-7B	7B	50·9	47·2–54·1	6·1	AX-95194687	–0·0058	17·3
RLD								
50–60cm	qRLD50-60.f-1B	1B	132·0	118·1–137·2	3·1	BS00022609	0·0705	8·7
50–60cm	qRLD50-60.f-2B	2B	5·8	2·1–7·5	3·1	AX-94505732	–0·0452	7·9
50–60cm	qRLD50-60.f-2D	2D	72·5	67·4–77·5	3·8	AX-94485593	–0·0566	12·4
50–60cm	qRLD50-60.f-5D	5D	35·5	29·9–40·8	3·1	AX-94774616	0·0483	7·9
60–70cm	qRLD60-70.f-6A	6A	30·9	30·3–33·4	3·2	AX-94579171	0·0633	9·4
60–70cm	qRLD60-70.f-7B	7B	11·7	4·0–21·0	5·1	AX-95652919	–0·0625	15·7
70–80cm	qRLD70-80.f-5D	5D	264·1	256·8–268·8	3·7	AX-94916991	–0·0287	9·7
RDW								
50–60cm	qRDW50-60.f-1A	1A	96·5	94·2–99·6	3·3	AX-94826839	0·0043	9·0
50–60cm	qRDW50-60.f-1B	1B	16·5	6·1–26·6	3·9	AX-94790297	–0·0045	14·2
50–60cm	qRDW50-60.f-2D	2D	70·5	60·8–78·6	3·2	AX-94485593	–0·0037	9·3
50–60cm	qRDW50-60.f-5D	5D	35·5	28·8–40·6	3·6	AX-94774616	0·0040	9·8
60–70cm	qRDW60-70.f-7A	7A	0·0	0·0–5·9	3·0	AX-94603119	–0·0039	10·8
70–80cm	qRDW70-80.f-5D	5D	267·1	263·4–269·2	4·2	AX-94916991	–0·0036	12·0
Diam								
50–60cm	qDiam50-60.f-2D	2D	25·9	22·7–39·6	5·2	AX-95102138	0·0058	14·8
50–60cm	qDiam50-60.f-5A	5A	196·2	185·6–201·3	3·1	AX-95235821	–0·0044	8·3

QTL names represent trait abbreviation, environment (f, field), a hyphen (-) and linkage group in which it is located.

Positive additive effects are from the Shamrock parent and negative effects are from the Shango parent.

Three QTLs associated with average RLD between 50 and 80cm were identified on chromosomes 5D, 6B and 7B (Table 1). In terms of variation explained, the QTL with the greatest positive effect on RLD was on 6B (0·0431cm cm^−3^ additive effect, 13·9 % phenotypic variation explained) where the Shamrock allele had a positive effect. Shamrock was also the high value allele for the QTL on 5D, whereas Shango contributed the high value allele for the QTL identified on 7B (–0·0342cm cm^−3^, 8·8 % phenotypic variation explained), in addition to a more significant QTL further down the linkage group for RDW (–0·0058mg cm^−3^, 17·3 % phenotypic variation explained). A second QTL for RDW was identified on chromosome 2A, with Shango also contributing the high value allele.

Due to the significant interacting effect on RLD and RDW of DH lines with depth, QTL analysis was also undertaken for the rooting traits within each 10cm soil layer between 50 and 80cm (Table 1). QTLs that explained variation in the population for average RLD were also present in the individual 10cm depths. This included the QTL on the short arm of 5D, contributed by a high value allele from Shamrock, for both RLD and RDW within the 50–60cm layer. Additionally, the QTL on 7B, contributed by a high value allele from Shango, was identified for RLD within the 60–70cm layer (Table 1); the peak markers for both these QTLs differed but the confidence intervals overlapped.

Co-locating QTLs were found for both RLD and RDW at multiple depths, with a QTL on 2D which explained variation in DH lines for RLD and RDW within the 50–60cm layer, with Shango contributing the high value allele. Additionally, within the 70–80cm layer, a QTL was identified on the long arm of 5D that explained variation in both RLD and RDW, with the high value allele contributed from Shango. Further QTLs were identified for RLD within the 50–60cm layer on the long arm of 1B, with a high value allele coming from Shamrock and the QTL on 2BS mentioned previously. Two QTLs were found to explain variation within the DH lines for RLD in the 60–70cm layer on 6A and 7B, with high value alleles contributed from Shamrock and Shango, respectively.

For RDW in the 50–60cm layer, additional QTLs were identified on 1A and the long arm of 1B, with high value alleles being contributed from both Shamrock and Shango, respectively. A single QTL explained variation in the population for RDW within the 60–70cm layer on the short arm of 7A, contributed by a high value allele from Shango. Only QTLs within the 50–60cm layer were identified for variation in root diameter; these comprised a QTL on the short arm of 2D, contributed by a high value allele from Shamrock, and the long arm of 5A, contributed by a high value allele from Shango.

### Non-glaucous doubled haploid lines had greater rooting at depth in the rhizotrons at tillering

**Fig. 3.**
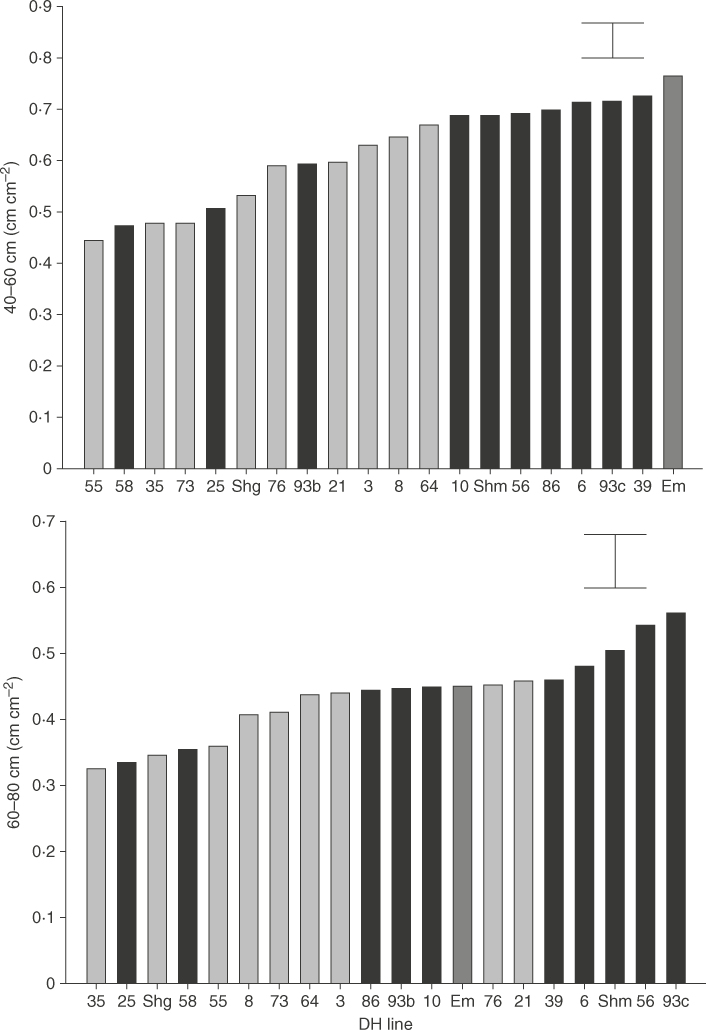
Root length of doubled haploid (DH) lines from Shamrock (Shm) × Shango (Shg) and a wild emmer accession (Em, dark shading). Glaucous DH lines are indicated by shaded columns and non-glaucous DH lines are indicated by hatched columns. Values derived from acetate tracings at 40–60 and 60–80cm soil layers on rhizotrons. Error bar is 1 SED.

**Fig. 4.**
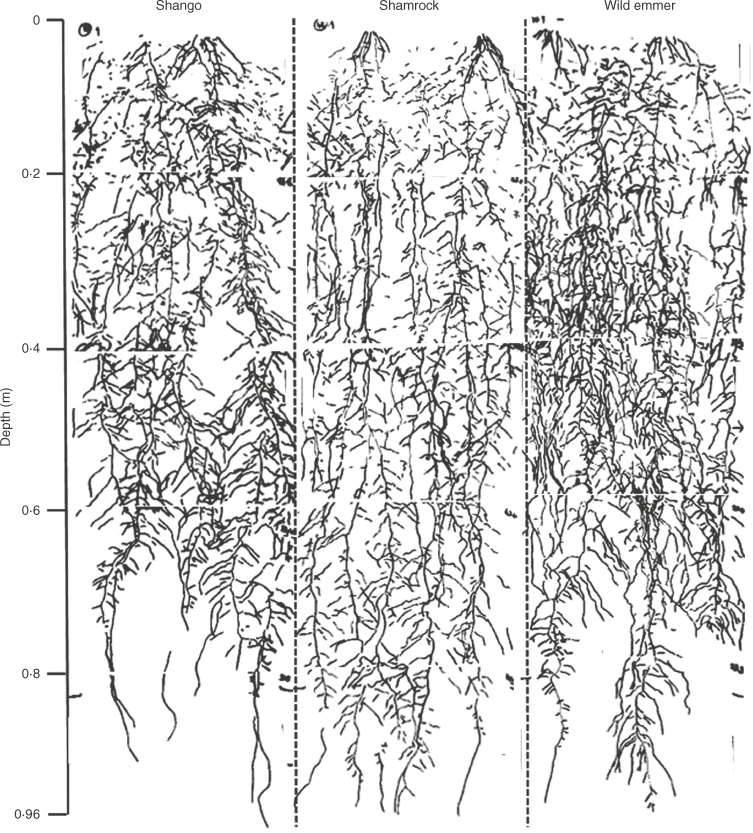
Full profile acetate tracings from a replicate rhizotron of Shango, Shamrock and a wild emmer accession (labelled), 45 d after sowing. Dashed lines separate each replicate rhizotron profile.

The RLD (cm cm^−3^) of roots separated from the rhizotron soil correlated significantly (*r*=0·82 *P*<0·001) with area-based root length (cm cm^−2^) measured on the acetate sheets. Consequently, only root length results from the acetate tracings are reported hereafter. Selected DH lines differed significantly (*P*<0·001) for root length in the 40–60 profile (Fig. 3; full profiles are shown in Supplementary Data Fig. S1). As in the field, Shamrock had greater root lengths at depth compared with Shango, significantly so (*P*<0·05) at 40–60cm (Figs 3 and 4). The larger rooting system of Shamrock was further confirmed in the dry weight distributions (Table 2), with Shamrock having significantly greater RDW compared with Shango in the 40–60 and 60–80cm layers, as well as the average in the whole profile. Shamrock had a greater shoot dry weight compared with Shango, but this was not significant (*P* > 0·05); no significant differences in root:shoot ratios were found between the DH lines.

**Table 2.** The effect of different genotypes and genotype groups on shoot and root characteristics in different depth ranges in the rhizotrons

Lines	Shoot dry matter (g)	Root dry weight (mg cm^−3^)			Mean root diameter (mm)		
		0–96cm	40–60cm	60–80cm	20–40cm	40–60cm	60–80cm
Shamrock	3·21	0·084	0·078	0·038	0·39	0·36	0·36
Shango	2·66	0·049	0·046	0·021	0·36	0·69	0·41
SED	0·346	0·0171*	0·0157*	0·0074*	0·057	0·115*	0·036
Non-glaucous	3·06	0·072	0·062	0·034	0·39	0·38	0·38
Glaucous	2·95	0·064	0·060	0·028	0·40	0·44	0·40
SED	0·134	0·0061	0·0060	0·0026*	0·018	0·036	0·011
Wild emmer	2·69	0·055	0·053	0·021	0·30	0·29	0·32
Doubled haploid mean	3·01	0·068	0·061	0·031	0·39	0·41	0·39
SED	0·306	0·0139	0·0014*	0·0059	0·041*	0·082	0·026*

SED

* *P* < 0 ·05.

The DH lines that exhibited the non-glaucous trait after flag leaf emergence in the field had, on average, increased rooting at depth, for both root length and RDW in the rhizotrons before stem extension (Table 2). Mean root lengths of non-glaucous lines were 0·649 and 0·458cm cm^−2^ in the 40–60 and 60–80cm soil layers, respectively; significantly (*P*<0·05) higher than glaucous lines where average root lengths were 0·563 and 0·405cm cm^−2^ (SED 0·0282 and 0·0262 for the 40–60 and 60–80cm layers, respectively). The single accession of wild emmer used had particularly high root lengths between 20 and 60cm, and differed significantly (*P*<0·02) from the mean of the DH lines at these soil depths (Figs 3 and 4; Supplementary Data Fig. S1). Wild emmer also produced finer roots so the increased root length was therefore not reflected in greater root mass (Table 2).

Root length of DH lines grown in the rhizotrons to the late tillering growth stage did not show a strong relationship with RLDs in the field at anthesis. Genotypes 56 and 93c were high performing lines in the 40–80cm layer in the rhizotrons (Fig. 3) but ranked only 85th and 80th, respectively, in the 50–80cm soil layer in the field. Comparing average root lengths from the acetate tracings for the A and B allele SNP genotype calls for average field RLD (Table 1) also did not show a significant difference in rooting ability within the different layers of the rhizotrons (Table 3). However, the QTL identified on 2BS, in close proximity to that for glaucousness, which explained RLD in the 50–60cm soil layer in the field showed a significant association with root length at 40–60 and 60–80cm depths in the rhizotrons. The DH lines exhibiting the A allele from Shamrock had significantly greater root length at these depths, in contrast to that in the field where lines exhibiting the B allele from Shango had greater RLDs in the 50–60cm layer post-anthesis.

**Table 3.** Average root length (cm cm^−2^) from the acetate tracings at each depth for the A (Shamrock) and B (Shango) alleles present in the selected lines of QTLs for average RLD in the field (5D, 6B and 7B, Table 1) and allocation of RLD in the 50–60cm depth in the field (2BS, Table 1)

Depth (cm)	5D (cm cm^−2^)	6B (cm cm^−2^)	7B (cm cm^−2^)	2BS (cm cm^−2^)
0–20	A: 0·459 B: 0·474	A: 0·447 B: 0·475	A: 0·448 B: 0·477	A: 0·471 B: 0·458
SED	0·0195	0·0200	0·0203	0·0192
20–40	A: 0·645 B: 0·684	A: 0·661 B: 0·662	A: 0·668 B: 0·657	A: 0·669 B: 0·653
SED	0·0290	0·0297	0·0302	0·0290
40–60	A: 0·627 B: 0·582	A: 0·605 B: 0·610	A: 0·622 B: 0·598	A: 0·649 B: 0·563
SED	0·0292	0·0299	0·0304	0·0289*
60–80	A: 0·447 B: 0·414	A: 0·433 B: 0·420	A: 0·451 B: 0·420	A: 0·458 B: 0·405
SED	0·0268	0·0275	0·0280	0·0265*
80–100	A: 0·190 B: 0·185	A: 0·198 B: 0·182	A: 0·195 B: 0·182	A: 0·177 B: 0·200
SED	0·0238	0·0243	0·0248	0·0235

SED

* *P* < 0·05.

### Non-glaucous doubled haploid lines were associated with smaller seedling root systems

**Fig. 5.**
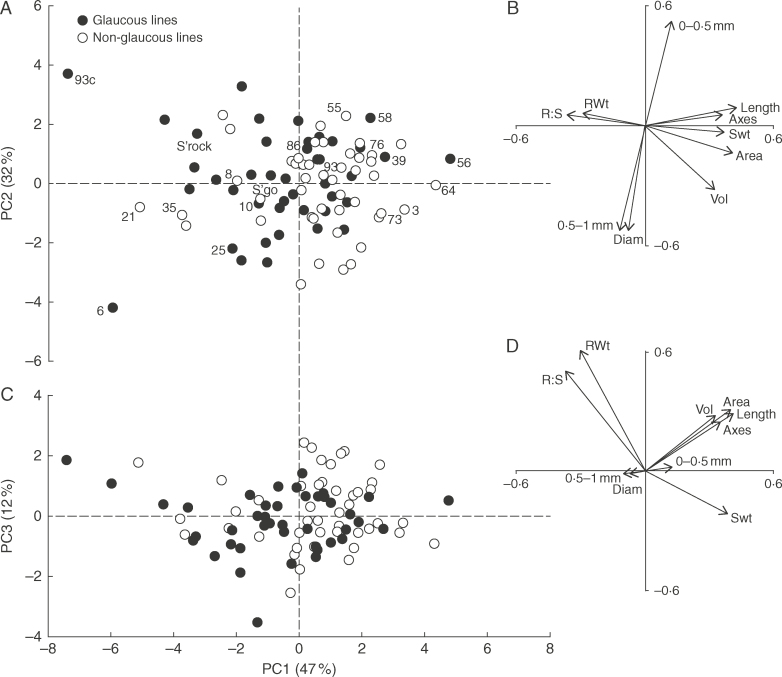
Line (Shamrock × Shango doubled haploid) scores (A, C) and vector loadings (B, D) from a principal components (PCs) analysis of seedling root traits. Area, root surface area; Axes, number of seminal axes; Diam, mean root diameter; Length, total root length; RWt, total root dry mass (DM); R:S, root:shoot mass ratio; SWt, total shoot mass (DM); Vol, total root volume; 0–0·5mm, proportion (%) of roots with a diameter of 0–0·5mm; 0·5–1mm, proportion (%) of roots with a diameter of 0·5–1mm. Point labels in (A) are the selected lines used in the rhizotron experiment.

Significant (*P*=0·001) differences occurred within the DH population for the root and shoot traits observed in the individual lines at the seedling stage (summarized in Table 4; detailed in Supplementary Data Table S1). Shamrock tended to have a smaller seedling root system, in terms of length, surface area and volume, than Shango, in contrast to root length measurements seen in the rhizotrons and in deeper soil in the field. However, as in the field and rhizotrons, Shamrock produced finer roots, having a significantly higher percentage of roots within the lower root diameter class 0–0·5mm (Table 4). The PCA captured 90 % of the variation within the Line × ten variate table (Supplementary Data Table S2) by three principal components (PCs; Fig. 5). Principal component 1 accounted for variation mostly in ‘size’ of the root system as measured by ‘WinRhizo’, i.e. number of root axes, root length and root surface area. Principal component 1 was also positively associated with shoot dry weight (and hence negatively associated with the root:shoot ratio) but negatively associated with root dry weight. Principal component 2 accounted for measures of root diameter, high values representing the finest roots. Principal component 3 mostly accounted for additional variation in root dry weight, not already accounted for by PC1 and showing less of an association with shoot dry weight.

**Table 4.** The effect of line from a doubled haploid progeny of Shamrock × Shango winter wheat on root and shoot characteristics when grown as seedlings in germination paper rolls

	Total root size					Root diameter			Shoot dry matter (mg)	Root:shoot (ratio)
	Length (cm)	Surface area (cm^2^)	Volume (cm^3^)	Dry matter (mg)	Seminal axes (no.)	Mean (mm)	0–0·5mm (%)	0·5–1mm (%)		
Parental lines										
Shamrock	57·6	7·82	0·085*	16·8	3·96	0·432	0·91*	0·08*	12·7	1·37
Shango	62·1	8·67	0·097*	16·9	4·00	0·448	0·87*	0·12*	13·2	1·34
Minimum value and responsible line among populations										
Minimum	51·0	7·18	0·075	13·6	3·75	0·417	0·77	0·05	10·7	0·88
Line (glaucous allele A/B)	6 (A)	93c (B)	93c (B)	10 (A)	18 (A)	93c (B)	6 (A)	93c (B)	93c (B)	64 (A)
Maximum value and responsible line among populations										
Maximum	72·3	9·86	0·110	19·6	4·72	0·476	0·94	0·23	16·3	1·94
Line (glaucous allele A/B)	56 (A)	56 (A)	72 (B)	93c (B)	64 (B)	6 (A)	93c (B)	6 (A)	64 (B)	93c (B)
SED	3·64	0·506	0·0062	1·19	0·206	0·0093	0·020	0·019	1·00	0·177
Pop. mean	62·7	8·73	0·097	16·2	4·27	0·444	0·89	0·11	14	1·21

SED

*P < 0 ·05 between Shamrock and Shango, *n* = 24.

Glaucousness QTL on 2BS, Shamrock is A allele and Shango is B.

The DH lines with the Shamrock allele for the glaucousness SNP marker (Table 1) had a negative association (*P*<0·05) with total root length and root surface area (*r*=–0·23 and –0·26, respectively), the opposite association to that seen in non-glaucous lines in the rhizotrons. The DH lines 93c and 6 had particularly short seedling roots (Table 4; Fig. 5) and fine and coarse roots, respectively. Both these lines, in contrast, performed well in the rhizotron experiment (Fig. 3); the relationships seen in the PCA and the negative relationship with the non-glaucous allele are still significant without these outliers.

**Fig. 6.**
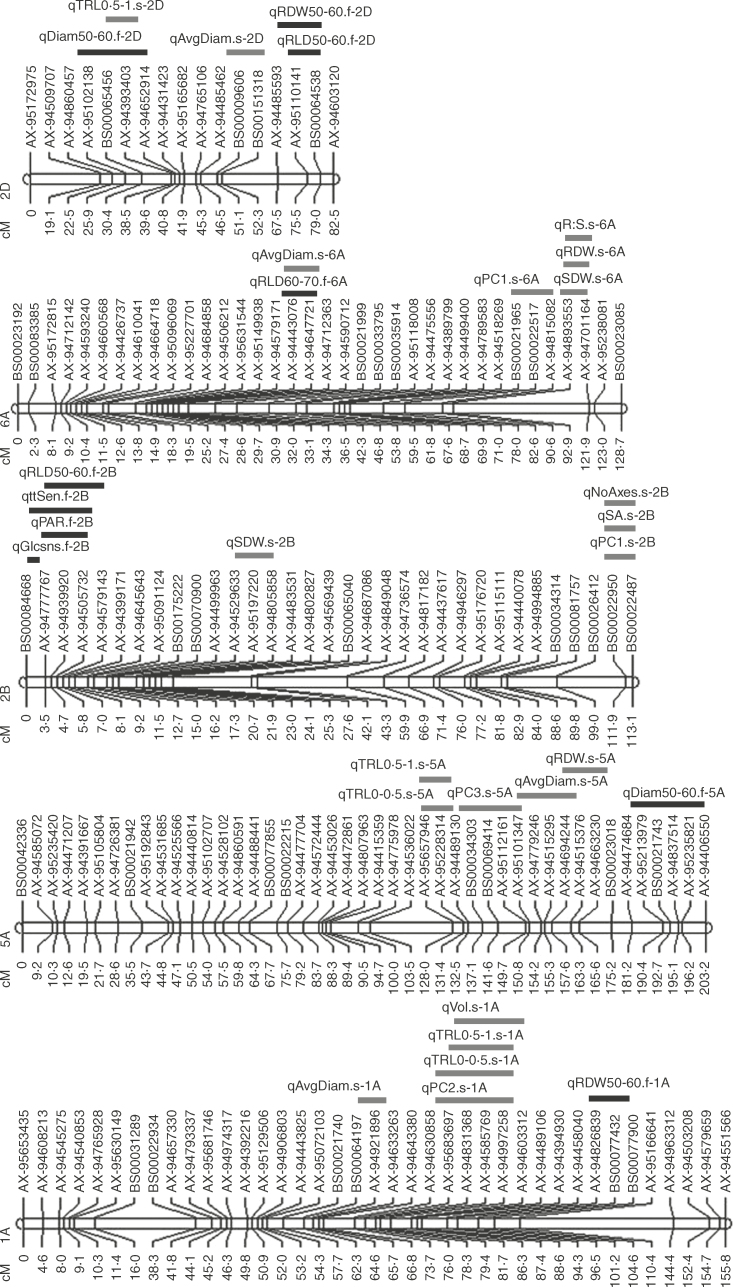
Diagram of six linkage groups (1A, 5A, 2B, 6A and 2D) of Shamrock × Shango with QTL locations. SNP marker names are labelled on the right of linkage groups and distance in centiMorgans is stated on the left of linkage groups. Only linkage groups with co-locating QTLs are shown. QTL locations and confidence intervals are given on bars to the right of linkage groups. QTLs are labelled, and black and grey bars represent field and seedling data, respectively.

The QTL analysis was completed for all the seedling root traits studied (Supplementary Data Table S3). However, here we focus on QTLs identified from the PC scores as they collate traits which explain overall variation in the root systems of the DH population (Okamoto *et al.*, 2012; Bouchet *et al.*, 2016). Three QTLs were evident for seedling root size (PC1; Table 5), with Shango contributing the positive allele for QTLs on 2B and 6A and Shamrock contributing a positive allele for the QTL identified on 4A. Shamrock contributed the majority of high value alleles for finer root diameter (PC2) with QTLs on 1A, 1D and 5B, and Shango contributed a high value allele on 7A. Three QTLs were identified for PC3, with Shango contributing positive alleles for QTLs on 1B and 5A, and Shamrock contributing a positive allele for the QTL identified on 3A. These QTLs, as expected, also explained phenotypic variation in the individual root traits, specifically 2B for root surface area and number of axes, 1A for root volume and proportion of roots in the two diameter classes, and 5B and 7A for average root diameter (Fig. 6; Supplementary Data Table S3).

**Table 5.** Quantitative trait loci (QTLs) from a Shamrock × Shango doubled haploid population for principal component (PC) scores derived from seedling root traits

Chromosome	QTL	Position (cM)	Confidence interval (cM)	LOD	Peak marker	Additive effect	Variation explained (%)
PC1 ‘Root system size’							
2B	qPC1.s-2B	111 ·9	104 ·0–113 ·0	3 ·8	BS00022950	–0 ·772	12 ·2
4A	qPC1.s-4A	18 ·8	9 ·8–27 ·2	3 ·8	BS00065863	0 ·837	11 ·4
6A	qPC1.s-6A	81 ·0	73 ·1–87 ·4	4 ·5	BS00021965	–0 ·949	12 ·4
PC2 ‘Root diameter’							
1A	qPC2.s-1A	76 ·0	73 ·5–81 ·8	3 ·3	AX-95683697	0 ·459	8 ·5
1D	qPC2.s-1D	49 ·1	39 ·0–50 ·1	3 ·5	AX-94413085	0 ·469	9 ·1
5B	qPC2.s-5B	128 ·0	122 ·4–139 ·1	3 ·0	BS00034333	0 ·442	8 ·0
7A	qPC2.s-7A	89 ·4	86 ·2–91 ·4	7 ·0	BS00077445	–0 ·630	13 ·7
PC3 ‘Root dry weight and size’							
1B	qPC3.s-1B	144 ·8	138 ·4–150 ·4	4 ·0	BS00071895	–0 ·360	9 ·9
3A	qPC3.s-3A	44 ·8	38 ·0–56 ·1	3 ·6	AX-94591588	0 ·347	7 ·9
5A	qPC3.s-5A	144 ·6	132 ·1–153 ·9	4 ·0	BS00069414	–0 ·374	9 ·6

QTL names represent trait abbreviation, environment (s, seedling), a hyphen (-) and linkage group in which it is located.

Positive additive effects are from the Shamrock parent and negative effects are from the Shango parent.

There were very few common QTLs explaining variation in both field and seedling root traits. However, there was some overlap of QTL confidence intervals and co-located QTLs identified for root traits within the 10cm soil layers in the field and seedling root traits (Fig. 6; Supplementary Data Table S3). The confidence intervals for the QTL identified on 2D for proportion of root length in the higher diameter class of 0·5–1mm and the QTL explaining variation in field root diameter in the 50–60cm layer did overlap, and the peak markers for the traits were neighbouring. The QTL identified on 6A for average seedling root diameter and field RLD in the 60–70cm layer co-located on the short arm of 6A. Shamrock contributed the high value alleles for these related QTLs.

## DISCUSSION

The experiments described here were primarily designed to investigate the potential association between the non-glaucous trait exhibited by Shamrock and RLDs at depth in the field, at mature growth stages susceptible to drought. The existence of such a link would indicate that the wild emmer introgression in the breeding of Shamrock contributes to the cultivar’s greater RLDs at depth. We confirm that RLDs at depth in commercially relevant field conditions in the UK (Ford *et al.*, 2006) were considerably smaller during grain fill than those reported in previous studies (Barraclough and Leigh, 1984; Gregory *et al.*, 1978a). Increasing rooting at depth is important to improve the acquisition of solutes and sub-soil water to increase resource use efficiency in future wheat cultivars (Foulkes *et al.*, 2009; Atta *et al.*, 2013). Multiple studies have identified the inadequacy of rooting at depth in UK wheat (Hoad *et al.*, 2004; White *et al.*, 2015), below the defined RLD of 1cm cm^−3^, thought to be sufficient for water uptake (van Noordwijk, 1983; Barraclough *et al.*, 1989; King *et al.*, 2003).

This study adds to the previously available evidence that Shamrock can have greater RLDs in deeper soil horizons compared with other UK-adapted wheats at critical periods of development (Gregory *et al.*, 2005; Ford *et al.*, 2006). Non-glaucous lines exhibited delayed senescence and greater light interception over the growing season, associated with a QTL on 2BS, co-locating with glaucousness. We have also demonstrated that a significant variation in RLD and RDW at depth exists in field-grown DH progeny of Shamrock × Shango, with the identification of QTL explaining variation in rooting density in the field post-anthesis; to our knowledge, this has not been previously achieved. However, the limited size of the DH population used in this study may have reduced QTL detection; this can be improved in future by increasing the number of lines studied.

Through assessing root phenotypic traits at mature growth stages in the field, we found an association between the non-glaucous trait mapped to the short arm of 2B (Simmonds *et al.*, 2008) and allocation of root length at different depths within the 50–80cm soil horizon. Shango achieved a higher RLD in the upper horizon and contributed the high value allele for a QTL identified on 2BS, for root allocation in this soil layer. In contrast, selected DH lines in the rhizotron experiment, with the Shamrock allele for this RLD QTL on 2BS, had significantly higher root length at the 40–80cm soil depths. This was in an environment where water supply was controlled and the soil profile was not allowed to dry out, a factor which may have affected root allocation. Additionally, the non-glaucous trait was associated with significantly (*P*<0·05) greater grain mass in the 2013/2014 season and a non-significant trend in the 2014/2015 season. The influence of the non-glaucous trait may then vary seasonally for its effects on rooting and also yield traits, due to a genotype × environment interaction (Acuna and Wade, 2012).

Shango and glaucous DH lines had greater seedling root length and surface area in the seedling screen compared with Shamrock and non-glaucous lines, with Shango contributing a high value allele for a QTL identified on the long arm of 2B for root system size. Other wheat seedling root screens have identified QTLs explaining root size traits on the long arm of 2B. QTLs which explain variation in wheat seedling total root length in both DH and RIL populations of durum and bread wheat were identified on the long arm of 2B by Kabir *et al.* (2015) and Maccaferri *et al.* (2016). In addition to root length, QTLs for root number were also identified in multiple studies on the long arm of 2B (Liu *et al.*, 2013; Zhang *et al.*, 2013; Maccaferri *et al.*, 2016). This consensus with the literature indicates the importance of the genetic influence of 2BL on seedling root size.

Root phenotypic traits, specifically root length and dry mass, of Shamrock × Shango DH lines grown in the rhizotrons and the seedling screen were not indicative of observations from field-grown mature plants. Lines which produced the least root length in the seedling screen were among those with the greatest root lengths in the rhizotrons, and selected lines which performed best in the rhizotrons had the lowest average RLD below 50cm in the field. Highly significant effects of growing conditions on root system size and morphology of cereal genotypes were also found in other studies. For example, root length was found to be significantly greater in gel media compared with soil, possibly due to reduced penetration resistance and nutrient concentration (Hargreaves *et al.*, 2009; Wojciechowski *et al.*, 2009). Root length traits differed significantly between sites and seasons in a study comparing Indian and Australian wheat cultivars in different regions in the two countries. The difference was attributed to soil heterogeneity, weather patterns and management on rooting traits down the soil profile (Rich *et al.*, 2016). Further, genotype effects also seem to interact with growth stage of wheat plants. Watt *et al.* (2013) found that root length of spring wheat seedlings correlated with the field up to the five leaf stage, but not at anthesis. Additional studies found weak genotypic correlations for root traits between early and later growth stages, specifically between stem elongation and heading and between mid-tillering and maturity (Motzo *et al.*, 1993; Ehdaie *et al.*, 2016). Specifically, the present study compared lines at multiple growth stages, grown in two different controlled environments, in addition to two field seasons with varying weather patterns.

Nonetheless, in the rhizotron experiment presented here, Shamrock did have greater root length at depth compared with Shango, an observation consistent with that found in the field in the 60–80cm depths. Root length of Shamrock was distributed more evenly throughout the profile, possibly associated with straighter seminal axes and more uniform branching in each soil layer. Shango had more tortuous seminal axes with longer root branches, which may have caused less proliferation below 60cm depth. Narrow seminal axes in wheat have been identified as a trait which increases rooting at depth due to reduced horizontal root growth and a more compact root system (Manschadi *et al.*, 2013). Shamrock also consistently had finer roots than Shango in the seedling, rhizotron and field experiments, with Shamrock mostly contributing the additive effects for QTLs explaining smaller root diameter in the seedling screen. Root diameter was negatively associated with root length in the DH lines at the different growth stages. Producing roots of a smaller diameter reduces the metabolic cost of the roots, thereby allowing more root biomass production and proliferation (Lynch, 2013). This aids resource uptake which is more closely related to root length and surface area than root mass (Eissenstat, 1992).

The co-location of QTLs for root traits in the seedling stage and mature roots in the field was associated with root diameter. This suggests that diameter is a more heritable trait than traits related to length. Root diameter is one of the few traits which have been bred for in wheat roots, with Richards and Passioura (1989) reducing xylem diameter in Australian cultivars to increase axial resistance of water flow to the roots to ensure water is not used up before critical growth periods.

## CONCLUSIONS

The specific location of the wild emmer introgression into Shamrock is unknown. Therefore, the positive effect of the identified QTLs on average RLD at depth in the field may be a contribution from wild emmer, although this cannot be proved at this stage. Similarly, we have not proved with confidence that the known emmer introgression on 2BS is responsible for Shamrock’s improved rooting at depth below the plough layer, due to non-glaucous lines having smaller seedling root size and the glaucous parent Shango contributing the high value allele for a QTL on 2BS, which explained higher rooting in the field at 50–60cm depth. The QTLs identified for rooting at depth in this study are based on a single field season; the inferences drawn would be strengthened by repetition across seasons and sites. The genetic diversity of rooting traits within the Shamrock × Shango population in different environments and growth stages is important for the further study of genetic controls on root architecture traits (Wasson *et al.*, 2012) which improve rooting at depth in the field at decisive growth stages. Therefore, continued study of this population and the association of the QTL on 2BS would be worthwhile in additional seasons and in environments where drought during grain filling can be controlled.

## SUPPLEMENTARY DATA

Supplementary data are available online at https://academic.oup.com/aob and consist of the following. Figure S1: root length of selected DH lines in the rhizotron profile. Table S1: average root and shoot characteristic values for each DH line measured in the seedling screen. Table S2: correlation coefficients between seedling variates using the means of Shamrock × Shango DH lines. Table S3: quantitative trait loci for seedling root traits in the DH population

## Supplementary Material

Supplementary_Information
