# Supplementary material for: Quantifying rooting at depth in a wheat doubled haploid population with introgression from wild emmer
Source: Ann Bot. 2017 Jun 26;120(3):457–70. doi: 10.1093/aob/mcx068 (PMC5591426; doi:10.1093/aob/mcx068)
Supplement: Supplementary_Information [file mcx068_suppl_Supplementary_Information.docx]

Supplementary Figure S1. Root length density (RLD) of selected doubled haploid lines from Shamrock (filled circles) x Shango (blank circles) and a wild emmer accession (filled square symbol). Average of three replicates per genotype. Glaucous doubled haploid lines are blank triangles and non-glaucous doubled haploid lines are filled triangles. Values derived from acetate tracings for the root profile of lines grown in rhizotrons. Error bar is 1 SED.


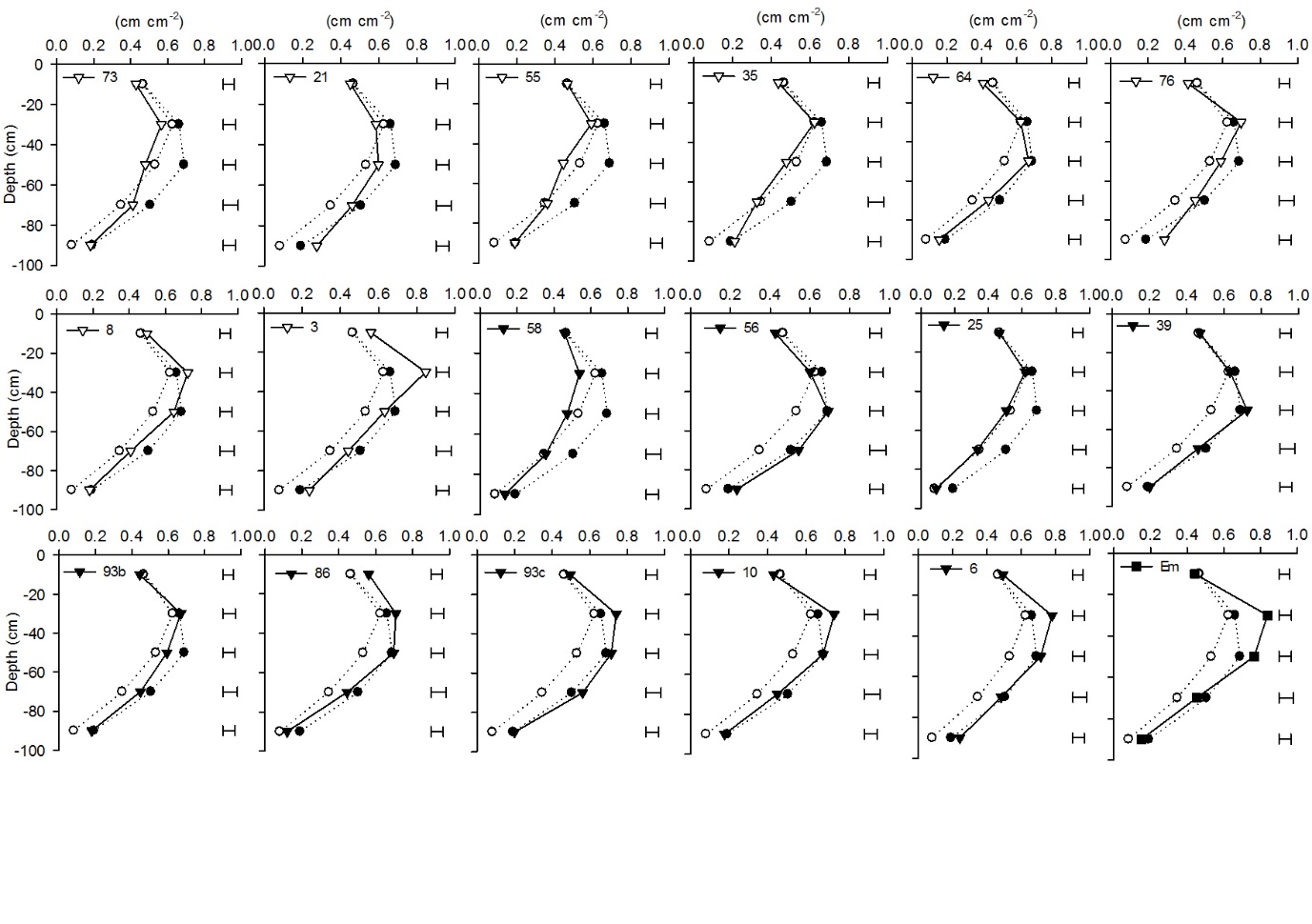


| Line  Supplementary Table S1. The effect of line from a doubled haploid progeny of Shamrock x Shango winter wheat on root and shoot characteristics when grown as seedlings in germination paper rolls, n=24. | Non-glaucous | Total root size | | | | |  | Root diameter | | | Shoot dry matter (mg) | Root: Shoot (ratio) |
| --- | --- | --- | --- | --- | --- | --- | --- | --- | --- | --- | --- | --- |
|  |  | Length (cm) | Surface area (cm^2^) | Volume (cm^3^) | Dry matter (mg) | Seminal Axes (no.) |  | Mean (mm) | 0-0.5 mm (%) | 0.5-1 mm (%) |  |  |
| Shm | Yes | 57.6 | 7.82 | 0.085 | 16.8 | 3.96 |  | 0.432 | 0.911 | 0.084 | 12.7 | 1.37 |
| Shng | No | 62.1 | 8.67 | 0.097 | 16.9 | 4.00 |  | 0.448 | 0.872 | 0.120 | 13.2 | 1.34 |
| 1 | No | 63.2 | 8.86 | 0.099 | 15.8 | 4.17 |  | 0.452 | 0.872 | 0.123 | 14.8 | 1.13 |
| 2 | No | 67.7 | 9.17 | 0.099 | 14.9 | 4.67 |  | 0.431 | 0.904 | 0.094 | 15.7 | 0.95 |
| 3 | No | 67.4 | 9.46 | 0.106 | 14.5 | 4.58 |  | 0.448 | 0.865 | 0.131 | 15.0 | 0.99 |
| 4 | No | 61.9 | 8.26 | 0.089 | 18.2 | 4.08 |  | 0.421 | 0.915 | 0.083 | 13.2 | 1.48 |
| 5 | No | 67.2 | 9.35 | 0.104 | 16.1 | 4.67 |  | 0.444 | 0.901 | 0.096 | 14.4 | 1.13 |
| 6 | Yes | 51.0 | 7.57 | 0.090 | 18.8 | 4.00 |  | 0.476 | 0.770 | 0.226 | 11.8 | 1.77 |
| 7 | Yes | 61.8 | 8.30 | 0.089 | 16.6 | 4.25 |  | 0.428 | 0.925 | 0.071 | 13.3 | 1.30 |
| 8 | No | 58.0 | 8.13 | 0.091 | 17.3 | 4.29 |  | 0.447 | 0.884 | 0.112 | 13.6 | 1.36 |
| 9 | No | 60.5 | 8.42 | 0.093 | 14.4 | 4.38 |  | 0.445 | 0.869 | 0.128 | 13.1 | 1.00 |
| 10 | Yes | 55.7 | 7.89 | 0.089 | 13.6 | 4.00 |  | 0.452 | 0.868 | 0.129 | 13.6 | 1.03 |
| 11 | Yes | 56.1 | 8.24 | 0.097 | 16.1 | 4.00 |  | 0.467 | 0.832 | 0.162 | 13.5 | 1.23 |
| 12 | Yes | 60.6 | 8.69 | 0.099 | 14.6 | 4.27 |  | 0.457 | 0.857 | 0.141 | 14.2 | 1.09 |
| 13 | No | 66.1 | 9.30 | 0.105 | 16.0 | 4.38 |  | 0.449 | 0.882 | 0.113 | 14.4 | 1.16 |
| 14 | No | 63.8 | 8.85 | 0.098 | 15.5 | 4.33 |  | 0.443 | 0.899 | 0.096 | 15.9 | 1.01 |
| 15 | No | 59.5 | 8.15 | 0.089 | 15.1 | 4.08 |  | 0.435 | 0.883 | 0.114 | 15.3 | 1.00 |
| 16 | No | 60.1 | 8.54 | 0.097 | 15.9 | 3.92 |  | 0.453 | 0.858 | 0.137 | 13.4 | 1.24 |
| 17 | No | 60.8 | 8.40 | 0.093 | 15.2 | 4.29 |  | 0.440 | 0.898 | 0.100 | 14.1 | 1.12 |
| 18 | Yes | 57.2 | 7.90 | 0.087 | 17.2 | 3.75 |  | 0.440 | 0.891 | 0.106 | 13.2 | 1.34 |
| 20 | No | 66.9 | 9.01 | 0.097 | 16.6 | 4.42 |  | 0.430 | 0.907 | 0.091 | 14.0 | 1.27 |
| 21 | No | 56.5 | 7.97 | 0.090 | 18.0 | 3.98 |  | 0.452 | 0.861 | 0.136 | 10.9 | 1.83 |
| 22 | Yes | 65.4 | 9.06 | 0.100 | 16.4 | 4.33 |  | 0.443 | 0.888 | 0.111 | 14.1 | 1.23 |
| 23 | No | 65.6 | 9.16 | 0.102 | 17.1 | 4.50 |  | 0.443 | 0.894 | 0.102 | 13.1 | 1.49 |
| 24 | No | 60.8 | 8.88 | 0.104 | 16.1 | 4.33 |  | 0.465 | 0.830 | 0.166 | 14.6 | 1.15 |
| 25 | Yes | 55.6 | 8.13 | 0.095 | 16.5 | 4.04 |  | 0.464 | 0.840 | 0.156 | 13.6 | 1.26 |
| 26 | No | 63.5 | 8.75 | 0.096 | 14.5 | 4.42 |  | 0.439 | 0.915 | 0.084 | 14.8 | 0.99 |
| 27 | No | 60.7 | 8.14 | 0.087 | 15.6 | 4.13 |  | 0.431 | 0.915 | 0.079 | 12.1 | 1.37 |
| 28 | No | 65.8 | 8.92 | 0.097 | 17.5 | 4.17 |  | 0.433 | 0.884 | 0.114 | 14.5 | 1.25 |
| 29 | No | 62.7 | 8.58 | 0.094 | 14.4 | 4.46 |  | 0.436 | 0.920 | 0.078 | 13.2 | 1.12 |
| 30 | No | 65.3 | 9.09 | 0.101 | 16.7 | 4.67 |  | 0.442 | 0.894 | 0.104 | 13.3 | 1.34 |
| 35 | No | 55.5 | 7.91 | 0.090 | 17.8 | 3.83 |  | 0.458 | 0.865 | 0.128 | 13.3 | 1.48 |
| 37 | No | 66.4 | 9.10 | 0.100 | 15.8 | 4.25 |  | 0.434 | 0.890 | 0.108 | 14.5 | 1.15 |
| 39 | Yes | 66.5 | 9.08 | 0.099 | 15.1 | 4.67 |  | 0.436 | 0.898 | 0.100 | 15.4 | 1.00 |
| 41 | Yes | 62.9 | 8.97 | 0.102 | 15.1 | 4.33 |  | 0.456 | 0.855 | 0.141 | 14.6 | 1.05 |
| 42 | Yes | 60.3 | 8.66 | 0.099 | 16.7 | 4.29 |  | 0.459 | 0.848 | 0.148 | 13.5 | 1.26 |
| 43a | Yes | 61.2 | 8.87 | 0.103 | 16.6 | 3.88 |  | 0.462 | 0.834 | 0.161 | 13.2 | 1.31 |
| 43b | Yes | 64.1 | 9.06 | 0.102 | 16.8 | 4.29 |  | 0.451 | 0.869 | 0.127 | 14.5 | 1.18 |
| 43c | No | 62.8 | 8.96 | 0.102 | 15.9 | 4.31 |  | 0.455 | 0.869 | 0.124 | 13.4 | 1.20 |
| 43d | Yes | 65.0 | 8.71 | 0.093 | 17.6 | 4.29 |  | 0.427 | 0.925 | 0.073 | 14.7 | 1.27 |
| 45 | Yes | 65.5 | 8.93 | 0.097 | 15.6 | 4.58 |  | 0.436 | 0.910 | 0.086 | 15.0 | 1.05 |
| 45b | Yes | 60.3 | 8.36 | 0.093 | 17.0 | 4.33 |  | 0.442 | 0.881 | 0.115 | 13.6 | 1.44 |
| 47 | Yes | 63.3 | 8.55 | 0.092 | 15.4 | 4.29 |  | 0.431 | 0.910 | 0.089 | 14.8 | 1.08 |
| 51 | Yes | 63.9 | 8.68 | 0.094 | 16.2 | 4.17 |  | 0.434 | 0.918 | 0.080 | 14.7 | 1.14 |
| 52 | Yes | 59.9 | 8.40 | 0.094 | 15.7 | 4.08 |  | 0.450 | 0.884 | 0.115 | 14.9 | 1.07 |
| 53 | No | 60.1 | 8.93 | 0.106 | 15.8 | 3.96 |  | 0.472 | 0.830 | 0.162 | 14.0 | 1.14 |
| 55 | No | 68.7 | 9.21 | 0.099 | 17.3 | 4.67 |  | 0.427 | 0.937 | 0.061 | 13.9 | 1.27 |
| 56 | Yes | 72.3 | 9.86 | 0.107 | 14.7 | 4.71 |  | 0.434 | 0.907 | 0.090 | 15.6 | 0.96 |
| 57 | No | 64.4 | 9.01 | 0.101 | 16.2 | 4.17 |  | 0.446 | 0.882 | 0.115 | 15.4 | 1.07 |
| 58 | Yes | 68.8 | 9.20 | 0.098 | 15.6 | 4.63 |  | 0.425 | 0.930 | 0.068 | 14.2 | 1.11 |
| 59 | No | 61.6 | 8.54 | 0.094 | 15.2 | 4.21 |  | 0.439 | 0.910 | 0.088 | 13.7 | 1.12 |
| 60 | Yes | 58.9 | 7.74 | 0.081 | 15.6 | 4.17 |  | 0.418 | 0.935 | 0.061 | 13.8 | 1.17 |
| 62 | No | 55.0 | 7.89 | 0.090 | 17.6 | 3.88 |  | 0.458 | 0.856 | 0.143 | 12.3 | 1.26 |
| 63 | Yes | 61.3 | 8.53 | 0.095 | 16.8 | 4.00 |  | 0.443 | 0.898 | 0.100 | 14.1 | 1.22 |
| 64 | No | 67.8 | 9.42 | 0.105 | 14.1 | 4.71 |  | 0.444 | 0.890 | 0.109 | 16.3 | 0.88 |
| 65 | Yes | 64.1 | 9.02 | 0.101 | 16.5 | 4.38 |  | 0.450 | 0.888 | 0.110 | 14.5 | 1.16 |
| 66 | No | 66.3 | 9.37 | 0.106 | 15.2 | 4.33 |  | 0.452 | 0.870 | 0.127 | 15.1 | 1.02 |
| 67 | Yes | 62.9 | 8.60 | 0.094 | 15.0 | 4.38 |  | 0.435 | 0.888 | 0.109 | 14.1 | 1.09 |
| 68 | Yes | 63.8 | 9.04 | 0.102 | 16.9 | 4.38 |  | 0.451 | 0.873 | 0.125 | 13.3 | 1.32 |
| 69 | Yes | 64.0 | 9.07 | 0.103 | 14.5 | 4.42 |  | 0.449 | 0.879 | 0.114 | 13.4 | 1.09 |
| 72 | No | 64.9 | 9.46 | 0.110 | 16.9 | 4.58 |  | 0.464 | 0.827 | 0.171 | 13.8 | 1.32 |
| 73 | No | 68.0 | 9.62 | 0.108 | 16.4 | 4.63 |  | 0.450 | 0.872 | 0.125 | 14.3 | 1.16 |
| 74 | No | 64.5 | 9.09 | 0.102 | 15.8 | 4.04 |  | 0.449 | 0.874 | 0.123 | 15.1 | 1.07 |
| 75 | No | 65.1 | 9.44 | 0.109 | 15.7 | 4.17 |  | 0.462 | 0.838 | 0.159 | 14.3 | 1.14 |
| 76 | No | 69.2 | 9.43 | 0.103 | 15.2 | 4.52 |  | 0.435 | 0.909 | 0.089 | 13.6 | 1.16 |
| 77 | Yes | 58.2 | 7.96 | 0.087 | 15.7 | 4.00 |  | 0.440 | 0.865 | 0.130 | 12.1 | 1.23 |
| 78 | Yes | 57.1 | 7.68 | 0.082 | 18.1 | 4.00 |  | 0.428 | 0.915 | 0.081 | 12.3 | 1.52 |
| 79 | Yes | 64.2 | 8.87 | 0.098 | 17.2 | 4.33 |  | 0.439 | 0.910 | 0.089 | 15.0 | 1.20 |
| 80 | No | 66.9 | 9.29 | 0.103 | 17.4 | 4.29 |  | 0.444 | 0.900 | 0.097 | 14.7 | 1.31 |
| 81 | Yes | 57.6 | 8.10 | 0.091 | 17.8 | 3.81 |  | 0.447 | 0.886 | 0.112 | 12.4 | 1.41 |
| 82 | No | 66.7 | 9.17 | 0.101 | 15.0 | 4.38 |  | 0.440 | 0.912 | 0.084 | 14.9 | 1.03 |
| 83 | Yes | 60.3 | 8.54 | 0.097 | 16.4 | 4.08 |  | 0.452 | 0.818 | 0.178 | 13.8 | 1.25 |
| 86 | Yes | 65.6 | 8.93 | 0.097 | 16.5 | 4.17 |  | 0.433 | 0.910 | 0.087 | 14.2 | 1.31 |
| 86a | Yes | 61.7 | 8.71 | 0.098 | 16.1 | 4.02 |  | 0.451 | 0.879 | 0.111 | 13.7 | 1.21 |
| 87 | No | 67.2 | 9.17 | 0.100 | 16.7 | 4.46 |  | 0.436 | 0.904 | 0.093 | 15.4 | 1.14 |
| 88 | Yes | 61.4 | 8.74 | 0.100 | 17.0 | 4.29 |  | 0.453 | 0.871 | 0.121 | 13.7 | 1.37 |
| 89 | No | 62.6 | 9.03 | 0.104 | 16.4 | 4.67 |  | 0.457 | 0.850 | 0.146 | 14.2 | 1.18 |
| 92b | No | 64.9 | 8.75 | 0.095 | 16.9 | 4.67 |  | 0.427 | 0.911 | 0.086 | 14.2 | 1.24 |
| 93a | No | 64.4 | 9.20 | 0.105 | 17.8 | 4.46 |  | 0.454 | 0.897 | 0.125 | 13.6 | 1.35 |
| 93b | Yes | 66.2 | 9.12 | 0.100 | 15.9 | 4.33 |  | 0.441 | 0.892 | 0.100 | 12.9 | 1.17 |
| 93c | Yes | 55.1 | 7.18 | 0.075 | 19.6 | 3.85 |  | 0.417 | 0.942 | 0.053 | 10.7 | 1.94 |
| 94a | Yes | 59.8 | 8.40 | 0.094 | 16.0 | 4.38 |  | 0.447 | 0.894 | 0.103 | 14.0 | 1.20 |
| 94b | No | 64.5 | 8.84 | 0.097 | 16.4 | 4.04 |  | 0.438 | 0.901 | 0.095 | 14.7 | 1.16 |
| 97b | Yes | 65.5 | 9.11 | 0.101 | 15.9 | 4.33 |  | 0.444 | 0.905 | 0.093 | 14.9 | 1.10 |
| 116a | No | 64.3 | 9.28 | 0.107 | 15.0 | 4.29 |  | 0.461 | 0.853 | 0.144 | 14.4 | 1.05 |
| 116c | Yes | 60.8 | 8.30 | 0.090 | 17.1 | 4.29 |  | 0.435 | 0.909 | 0.088 | 14.1 | 1.24 |
| 119a | Yes | 64.2 | 8.67 | 0.094 | 15.2 | 4.42 |  | 0.432 | 0.907 | 0.091 | 14.6 | 1.08 |
| 119b | No | 66.7 | 9.06 | 0.098 | 14.0 | 4.50 |  | 0.434 | 0.919 | 0.080 | 13.5 | 1.08 |
| 119c | Yes | 58.5 | 8.20 | 0.092 | 17.1 | 3.96 |  | 0.445 | 0.873 | 0.124 | 13.8 | 1.29 |
| S.E.D. (616 d.f.) | | 3.64 | 0.506 | 0.006 | 1.19 | 0.206 |  | 0.009 | 0.02 | 0.019 | 1.00 | 0.177 |

Supplementary Table S2. Correlations (*r*, d.f. = 88; critical values for *P* = 0.05 and 0.01 are 0.21 and 0.28 respectively, highlighted) between seedling variates using means from 89 lines of a doubled haploid population from Shamrock x Shango winter wheat

| Variate | Mean root diameter | Roots 0-0.5 mm diameter (%) | Roots 0.5-1 mm diameter (%) | Total root length | Total root dry matter | Root: shoot ratio | Total root surface area | Total shoot dry matter | Number of seminal axes |
| --- | --- | --- | --- | --- | --- | --- | --- | --- | --- |
| Roots 0-0.5 mm diameter (%) | -0.91 |  |  |  |  |  |  |  |  |
| Roots 0.5-1 mm diameter (%) | 0.91 | -0.99 |  |  |  |  |  |  |  |
| Total root length | -0.31 | 0.39 | -0.38 |  |  |  |  |  |  |
| Total root dry matter | 0.00 | -0.07 | 0.06 | -0.37 |  |  |  |  |  |
| Root: shoot ratio | 0.01 | -0.11 | 0.10 | -0.54 | 0.85 |  |  |  |  |
| Total root surface area | 0.10 | 0.01 | -0.01 | 0.91 | -0.39 | -0.56 |  |  |  |
| Total shoot dry matter | -0.06 | 0.15 | -0.13 | 0.63 | -0.50 | -0.80 | 0.63 |  |  |
| Number of seminal axes | -0.21 | 0.27 | -0.26 | 0.76 | -0.39 | -0.45 | 0.71 | 0.49 |  |
| Total root volume | 0.47 | -0.34 | 0.34 | 0.68 | -0.34 | -0.48 | 0.92 | 0.53 | 0.55 |

| Trait  Supplementary Table S3. Quantitative trait loci (QTL) from a Shamrock x Shango doubled haploid population for seedling root traits. QTL names represent trait abbreviation, environment (s, seedling), a hyphen (-) and linkage group it is located. Positive additive effects are from Shamrock parent and negative from Shango parent. | QTL | Chromosome | Position (cM) | Confidence Interval (cM) | LOD | Peak Marker | Additive Effect | Variation explained (%) |
| --- | --- | --- | --- | --- | --- | --- | --- | --- |
| TRL | qTRL.s-2A | 2A | 221.8 | 217.8-226.6 | 4.2 | BS00081630 | -1.397 | 11.8 |
| SA | qSA.s-2A | 2A | 223.0 | 217.8-227.0 | 3.1 | BS00021693 | -0.152 | 7.6 |
| SA | qSA.s-2B | 2B | 111.9 | 103.8-113.0 | 3.0 | BS00022950 | -0.164 | 8.7 |
| Vol | qVol.s-1A | 1A | 78.3 | 76.2-85.0 | 7.1 | AX-94831368 | -0.0032 | 20.1 |
| AvgDiam | qAvgDiam.s-1A | 1A | 64.3 | 62.0-64.6 | 5.0 | BS00064197 | -0.0035 | 7.9 |
| AvgDiam | qAvgDiam.s-2D | 2D | 51.1 | 47.9-60.4 | 5.4 | BS00009606 | 0.0037 | 8.6 |
| AvgDiam | qAvgDiam.s-5A | 5A | 159.6 | 151.1-162.7 | 7.1 | AX-94694244 | -0.0045 | 12.2 |
| AvgDiam | qAvgDiam.s-5B | 5B | 131.0 | 124.5-139.9 | 4.7 | BS00034333 | -0.0036 | 8.0 |
| AvgDiam | qAvgDiam.s-6A | 6A | 30.9 | 29.1-32.5 | 7.4 | AX-94579171 | 0.0045 | 12.4 |
| AvgDiam | qAvgDiam.s-7A | 7A | 90.4 | 86.5-94.5 | 6.5 | BS00077445 | 0.0043 | 10.9 |
| %TRL 0-0.5 mm | qTRL0-0.5.s-1A | 1A | 76.0 | 73.8-81.8 | 4.1 | AX-95683697 | 0.0087 | 7.8 |
| %TRL 0-0.5 mm | qTRL0-0.5.s-5A | 5A | 128.0 | 116.2-131.5 | 4.3 | AX-95657946 | 0.0093 | 8.1 |
| %TRL 0-0.5 mm | qTRL0-0.5.s-5B | 5B | 128.0 | 120.2-137.5 | 3.2 | BS00034333 | 0.0081 | 6.2 |
| %TRL 0-0.5 mm | qTRL0-0.5.s-7A | 7A | 103.3 | 101.6-106.7 | 4.4 | AX-94498468 | -0.0095 | 8.2 |
| %TRL 0.5-1 mm | qTRL0.5-1.s-1A | 1A | 78.3 | 74.0-81.3 | 4.0 | AX-94831368 | -0.0080 | 7.0 |
| %TRL 0.5-1 mm | qTRL0.5-1.s-1D | 1D | 49.1 | 40.9-50.1 | 3.8 | AX-94413085 | -0.0078 | 6.6 |
| %TRL 0.5-1 mm | qTRL0.5-1.s-2D | 2D | 30.4 | 26.9-36.2 | 5.4 | BS00065456 | 0.0104 | 9.8 |
| %TRL 0.5-1 mm | qTRL0.5-1.s-5A | 5A | 123.5 | 113.3-131.7 | 5.6 | AX-94536022 | -0.0110 | 12.2 |
| %TRL 0.5-1 mm | qTRL0.5-1.s-5B | 5B | 124.0 | 117.8-132.4 | 6.1 | BS00034333 | -0.0107 | 11.4 |
| %TRL 0.5-1 mm | qTRL0.5-1.s-7A | 7A | 103.3 | 100.6-105.6 | 5.7 | AX-94498468 | 0.0105 | 10.5 |
| RDW | qRDW.s-5A | 5A | 165.6 | 160.1-175.1 | 3.8 | AX-94663230 | -0.3506 | 8.4 |
| RDW | qRDW.s-6A | 6A | 92.9 | 89.5-102.9 | 5.1 | AX-94893553 | 0.4179 | 11.9 |
| SDW | qSDW.s-2B | 2B | 17.3 | 16.3-19.9 | 3.7 | AX-94529633 | -0.7352 | 12.5 |
| SDW | qSDW.s-6A | 6A | 92.9 | 90.4-101.0 | 3.4 | AX-94893553 | -0.3439 | 9.6 |
| R:S | qR:S.s-6A | 6A | 92.9 | 90.7-103.4 | 3.2 | AX-94893553 | 0.0594 | 10.1 |
| No Axes | qNoAxes.s-2B | 2B | 111.9 | 104.3-113.0 | 3.7 | BS00022950 | -0.0855 | 10.8 |
| No Axes | qNoAxes.s-6B | 6B | 57.7 | 48.4-65.8 | 3.1 | AX-95082971 | -0.0848 | 11.0 |
| No Axes | qNoAxes.s-7A | 7A | 162.8 | 155.1-168.8 | 3.6 | BS00022169 | 0.0918 | 10.4 |

TRL; total root length, SA; surface area, Vol; root volume, AvgDiam; average diameter, %TRL 0-0.5 mm; percentage of root length in the 0-0.5 mm diameter class, %TRL 0.5-1 mm; percentage of root length in the 0.5-1 mm diameter class, RDW; root dry weight, SDW; shoot dry weight, R:S; root shoot ratio, No Axes; number of seminal axes
